# National Society

**Published:** 1859-10

**Authors:** 


### NATIONAL SOCIETY.

### REPORT OF THE COMMITTEE ON CONSTITUTION.

*Mr. Chairman and Gentlemen:*—The committee appointed by this Convention to draft a Constitution, beg leave to report, that they have attended to the duty assigned them, and would respectfully offer the following plan of organization; trusting that its imperfections and shortcomings may be remedied by *your reflections* and the *judgment of those* who shall participate in the final adoption of *a constitution* for the proposed association. A due regard for our own reputation, as well as a just appreciation of the rights of others, make it obligatory, that we should state, and desire the same to be placed upon record, that the plan of organization we submit to you, is based upon a constitution that was framed by some of the brightest and best minds of our country. We refer to the American Medical Association.

### PLAN OF ORGANIZATION.

### SEC. I.—NAME.

THIS organization shall be known by the name, style, and title of the *American Dental Association.*

### SEC. II.—OBJECTS.

The objects of this Association shall be, to cultivate the science and art of dentistry, and all its collateral branches, to elevate and sustain the professional character of dentists, to promote among them mutual improvement, social intercourse, and to collectively represent and have cognizance of the common interests of the dental profession in every part of the United States.

### SEC. III.—MEMBERS.

*The members* of this Association shall be exclusively practitioners of dentistry, holding their appointment to membership, either as *delegates* from local institutions, as *members by invitation*, or as *permanent members.*

*The delegates* shall receive their appointment from permanently organized dental societies and dental colleges in the Union, each delegate holding his appointment for one year, and until another is appointed to succeed him.

Each local society shall be entitled to send to the Association one *delegate* for every *five* of its active members, and the faculty of each college to send one of its members as a representative.

*The members by invitation* shall be practitioners of *reputable standing*, from sections of the country not otherwise represented at the meeting. They shall receive their appointment by invitation of the meeting after an introduction from any of the members present.

*The permanent members* shall consist of all those who have served in the capacity of delegates, and of such other members as may receive their appointment by unanimous vote.

Each delegate, member by invitation, and permanent member, shall be entitled to debate, and vote on all questions agitated in the associations, and be eligible to any office in the gift of the Association.

To defray the expense of printing the *transactions*, and to meet other incidental expenses, the sum of dollars shall be assessed annually upon all the members present at meetings of the Association. The payment of this assessment shall be required of those in attendance previous to taking their seats, and participating in the business of the session.

No member who is not present at a meeting of the Association, shall be required to pay the annual assessment; but no such member shall be entitled to a copy of the printed *transactions*, unless he pay into the treasury a sum of not less than the amount paid by members in attendance.

Each member elect, prior to the permanent organization of the annual meeting, or before voting on any question after the meeting has been organized, must sign these regulations, inscribing his name in full, specifying in what capacity he attends, and if a delegate, the title of the institution from which he has received his appointment.

No one shall be permitted to address the Association before giving his name and residence, which shall be distinctly announced from the chair.

SEC. IV.—MEETINGS.

The regular meetings of the Association shall be held annually, and commence on the third Tuesday in July. The place of meeting shall never be the same for any two years in succession, and shall be determined each year by vote of the Association.

SEC. V.—OFFICERS.

The officers of the Association shall be a President, two Vice-Presidents, Corresponding Secretary, Recording Secretary, and a Treasurer. They shall be nominated by a special committee of one member from every State represented at the meeting, and shall be elected by vote on general ticket. Each officer shall hold his appointment for one year, and until another is elected to succeed him.

*The President* shall preside at the meetings, and perform all the duties that custom, or parliamentary usage may require.

*The Vice-Presidents.*—In the absence of the President, one of the Vice-Presidents shall assume all the duties of the office; and, in the absence of these officers, a Chairman, *pro tem.* shall be appointed *viva voce.*

*The Corresponding Secretary* shall attend to the correspondence with the Association, and give due notice of the time and place of each annual meeting.

*The Recording Secretary* shall keep accurate minutes of the proceedings of the Association; preserve the archives and unpublished transactions, and attend to all other duties that appertain to the office.

*The Treasurer* shall have the immediate charge and management of the funds and property of the Association, and shall present a balanced account, duly authenticated, at every regular meeting, to the Committee of Publication.

SEC. VI.—STANDING COMMITTEES.

The following Standing Committees shall be organized at every annual meeting, to prepare, arrange, and expedite business for each ensuing year, and to carry into effect the orders of the Association not otherwise assigned, namely: a Committee of Arrangements, of Publication, on Prize Essays, on Scientific Investigation, on Dental Pathology and Surgery, on Mechanical Dentistry, and on Dental Education and Dental Literature.

*The Committee of Arrangements* shall be composed of five members, one of whom, if possible, shall reside in the place at which the Association is to hold its next ensuing annual meeting. They shall be required to procure suitable accommodations for the meeting: to verify and report upon the credentials of membership; to receive and announce all essays and memoirs voluntarily communicated, either by members of the Association, or by others through them; and to select and announce at the earliest period practicable after their appointment, subjects for discussion at the succeeding annual meeting.

*The Committee of Publication,* of which the Secretaries and Treasurer must constitute a part, shall be authorized to employ a competent reporter, to furnish an accurate report of the proceedings of each meeting. They shall superintend the publication and distribution of such portions of the Transactions as the Association may direct. The five members of this committee shall annul, accredit, and authenticate the accounts of the Treasurer, and present a statement of the same in the annual report of the committee; which report shall specify the character and cost of the publications of the Association during the year; the number of copies still at the disposal of the meeting, and the funds on hand for further operations. The committee shall be instructed to print at the beginning of each volume of the *Transactions*, the following disclaimer, viz.: *The American Dental Association*, although formally accepting and publishing the report of the various standing committees, and essays read before the Association, holds itself wholly irresponsible for the opinions, theories, or criticism therein contained, except when so decided by special resolution.

*The Committee on Prize Essays* shall consist of five members. Two prizes, of dollars each, shall be awarded to the best two communications reported on favorably by the committee, and directed by the Association to be published.

*The Committee on Scientific Investigation* shall be composed of six members, to be divided into two sections; one on the Physiological and Microscopical anatomy of the teeth, and one on Chemistry in its relations to Dentistry. It shall be the duty of the different sections to institute, during the period of their appointment, a series of investigations and experiments in these departments, and present to the Association the result of their labors.

*The Committee on Dental Pathology and Surgery*, consisting of five members, shall have under consideration everything that appertains to the pathological conditions of the teeth and adjacent tissues, and the remedial agencies embraced under the head of operative dentistry. All improvements in the latter department shall be thoroughly tested, and reported on before the Association by this committee.

*The Committee on Mechanical Dentistry* shall consist of five members, who shall receive and take cognizance of plans, improvements, and specimens in this department, that may be presented by persons desiring to bring them before the Association. The committee shall be authorized to reject those that they may deem unworthy of presentation.

*The Committee on Dental Education and Dental Literature*, consisting of five members, shall make annual reports on these important subjects.

The selection of the chairmen and members of the different committees, shall be referred to a nominating committee.

AMENDMENTS.

These articles may be altered and amended with the consent of three-fourths of the members present, at the next subsequent annual meeting to that at which such amendment or alteration may have been proposed.

In acknowledgment of having adopted the foregoing propositions, and of our willingness to abide by them, and use our endeavors to carry into effect the objects of this Association, as above set forth, we have hereunto set our names.

| NAMES OF MEMBERS. | RESIDENCE. | INSTITUTIONS REPRESENTED. |
| --- | --- | --- |
| | | |

THE ORDER OF BUSINESS.

The order of business at the annual meetings of the American Dental Association shall at all times be subjected to the vote of three-fourths of all the members in attendance; and, until permanently altered, except when for a time suspended, it shall be as follows:

1. The temporary organization of the meeting, preparatory to the election of officers.
2. The report of the committee of arrangements, on the credentials of members, after the latter have registered their names and addresses, and the titles of the institutions which they represent.
3. The calling of the roll.
4. The election of officers.
5. The reading of minutes.
6. The reception of members not present at the opening of the meeting, and the reading of notes from absentees.
7. The reception of members by invitation.
8. The reading and consideration of the stated annual reports from the standing committees.
9. The reading of essays.
10. The discussion of topics selected for the session.
11. The selection of the next place of annual meeting.
12. The new appointments to fill the standing committees.
13. Resolutions introducing new business, and instructions to the permanent committees.
14. Unfinished and miscellaneous business.
15. Adjournment.

*Committee.* { J. H. McQuillen, W. M. Wright, J. Richardson.

